# NDRG2 overexpression enhances glucose deprivation-mediated apoptosis in breast cancer cells via inhibition of the LKB1-AMPK pathway

**DOI:** 10.18632/genesandcancer.17

**Published:** 2014-05

**Authors:** Hak-Su Kim, Myung-Jin Kim, Jihyun Lim, Young Yang, Myeong-Sok Lee, Jong-Seok Lim

**Affiliations:** ^1^ Department of Biological Sciences and the Research Center for Women's Diseases, Sookmyung Women's University, Seoul, Republic of Korea

**Keywords:** NDRG2, AMPK, glucose deprivation, apoptosis

## Abstract

The newly identified tumor suppressor, N-myc downstream-regulated gene 2 (NDRG2), has been studied in various cancers because of its anticancer and antimetastasis effects. In this study, we examined the effect of NDRG2 expression on cell viability in MDA-MB-231 human breast cancer cells under conditions that are similar to the microenvironment of solid tumors, which include glucose deprivation. NDRG2 overexpression enhanced the pro-apoptotic effects of glucose deprivation. Glucose deprivation also induced the activation of AMP-activated protein kinase (AMPK), which plays a role in protecting tumor cells from metabolic stresses. NDRG2 overexpression strongly reduced glucose deprivation-induced AMPK phosphorylation and increased the cleavage of poly (ADP-ribose) polymerase (PARP), which indicated the induction of apoptosis. The expression of a constitutively active form of AMPK effectively blocked glucose deprivation-induced apoptosis in NDRG2-overexpressing MDA-MB-231 cells. Moreover, NDRG2 overexpression also enhanced the pro-apoptotic effects of 2-deoxyglucose (2-DG) or hypoxia, an inducer of metabolic stresses. Finally, we showed that LKB1 is an upstream kinase of AMPK that is involved in the inhibition of glucose deprivation-induced AMPK activity in NDRG2-overexpressing cells. Our findings collectively suggest that NDRG2 is a negative regulator of AMPK activity and functions as a sensitizer of glucose deprivation.

## INTRODUCTION

N-myc downstream-regulated gene 2 (NDRG2) is the second member of the NDRG family of genes that is involved in cell differentiation, proliferation, death, and migration [1,2]. Human NDRG2 was first identified in a normal human brain cDNA library and showed 57% sequence identity to NDRG1 and NDRG3 and 65% identity to NDRG4 [1]. Previous studies of NDRG2 function have included the following: anti-proliferation effects in tumor cells [3], differentiation into dendritic cells [4], and the induction of cell apoptosis [5]. NDRG2 was shown to inhibit the cancer cell metastasis through the attenuation of active TGF-β production [6] or through the suppression of nuclear factor κB activity [7]. Furthermore, NDRG2 attenuated tumor cell proliferation via the down-regulation of activator protein 1 (AP-1) activity in human colon carcinoma cells [8]. Moreover, the up-regulation of NDRG2 by oxygen-glucose deprivation (OGD) increased OGD-induced apoptosis in C6-originated astrocytes via the up-regulation of the p53 and Bax proteins [9]. Although evidence that supports anti-cancer and antimetastasis functions of the NDRG2 gene is expanding, NDRG2 function in the context of the metabolism of solid tumors remains uncertain.

According to the Warburg effect, cancer cells induce their metabolic changes to sustain proliferation and utilize glycolysis under normoxic conditions. A sufficient glucose supply facilitates rapid cell growth through the generation of intermediates that are required for the synthesis of essential cellular components [10,11]. However, solid tumors are exposed to microenvironments that are characterized by low levels of nutrients and oxygen because the tumors tend to outgrow the existing vasculature. To survive periods of metabolic stress, tumor cells must engage adaptive strategies. AMP-activated protein kinase (AMPK) plays a central role in the cellular sensing of energy availability and induces metabolic adaptation and cell survival [12]. AMPK exists as a heterotrimeric protein with an α -catalytic subunit and β -, and γ -regulatory subunits. The phosphorylation of Thr^172^ in the α-catalytic subunit is a critical event that is required for the activation of AMPK. Two major upstream kinases that are involved in the phosphorylation of Thr^172^ are the liver kinase B1 (LKB1) and the Ca^2+^/calmodulin-dependent protein kinase 2 (CaMKK2). The LKB1-AMPK pathway operates as an intracellular energy sensor and is activated during energy stress when the intracellular AMP/ATP ratio is elevated. The CaMKK2-AMPK pathway is usually elevated when intracellular Ca^2+^ levels are elevated [13,14]. Whereas AMPK hyper-activation is associated with anti-tumorigenic effects, multiple studies have indicated that physiological AMPK activation is pro-tumorigenic. In previous research that used an established rat brain tumor model, AMPK was strongly activated during the early stages of solid tumor formation [15]. AMPK activity is also involved in the resistance mechanisms that are induced by the anti-cancer agent cisplatin [16], and it has been reported that LKB1-null mouse embryonic fibroblasts (MEFs) are resistant to oncogene-induced transformation [17] and that H-Ras-transformed AMPKα1/α2-null MEFs are impaired in their ability to form tumors in an *in vivo* xenograft model [18]. In particular, glucose deprivation-induced AMPK activation has been shown to induce metabolic adaptation and cell survival in various cell types, including MEFs [18], pancreatic cancer cells [19], glioblastomas [20], colon cancer cells [21], and prostate cancer cells [22].

In this study, we investigated whether NDRG2 overexpression results in an increase in glucose deprivation-induced cell death in MDA-MB-231 cells. NDRG2 attenuated glucose deprivation-induced AMPK activity and performed a critical function in glucose deprivation-induced cell death. We also found that as an upstream regulatory kinase of AMPK, LKB1 is involved in the inhibition of glucose depletion-induced AMPK activity by NDRG2. In summary, NDRG2 is a negative regulator of AMPK activity and functions as a sensitizer to glucose deprivation.

## RESULTS

### NDRG2 overexpression exacerbates glucose deprivation-induced apoptosis in MDA-MB-231 cells

**Fig. 1:**
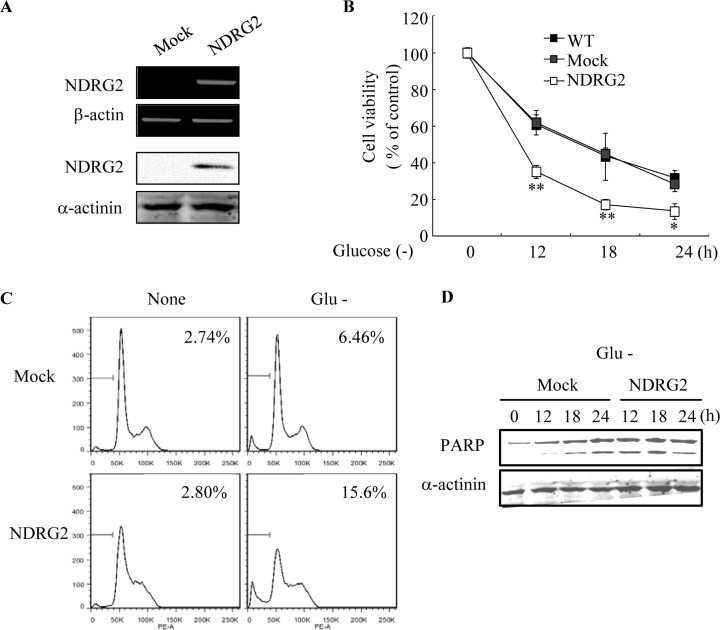
Effect of NDRG2 overexpression on glucose deprivation-mediated apoptosis in MDA-MB-231 cells (A) The expression levels of NDRG2 mRNA (upper panel) and protein (lower panel) in MDA-MB-231-mock (Mock) and MDA-MB-231-NDRG2-transfected cells (NDRG2) were examined by RT-PCR and western blot analysis. (B) MDA-MB-231-WT, -mock, and -NDRG2 cells were exposed to glucose deprivation for the indicated times. Cell viability was examined with the MTT assay. The results of three experiments are expressed as the mean ± SE. *p < 0.05 and **p < 0.01, compared with the MDA-MB-231-mock cells. (C) MDA-MB-231-mock and -NDRG2 cells were exposed to glucose deprivation for 18 h. Cells were collected, fixed in 70% ethanol, and stained with propidium iodide before FACS analysis. The percentage of sub-G1 DNA content is indicated. (D) MDA-MB-231-mock and -NDRG2 cells were exposed to glucose deprivation for the indicated times. Total cell extracts were prepared and subjected to western blot analysis using anti-PARP and anti-α -actinin antibodies.

To determine the effect of NDRG2 overexpression on glucose deprivation-induced cell death, we first established stable clones of MDA-MB-231 breast cancer cells following transfection with the pCMV-Taq-2B (mock) or pCMV-Taq-2B-NDRG2 (NDRG2) plasmids. After stable transfection, we determined the efficacy of cell death under both normal and glucose-deprived conditions. The level of NDRG2 mRNA in MDA-MB-231-NDRG2 cells was dramatically higher than in the MDA-MB-231-mock cells. The expression of the NDRG2 protein was also confirmed by western blot analysis (Fig. 1A). MDA-MB-231-wild type (wt), -mock, and -NDRG2 cells were exposed to glucose-free medium for the indicated periods of time, and cell viability was measured using MTT assay. MDA-MB-231-NDRG2 cells were found to be relatively sensitive to glucose deprivation-induced cytotoxicity and resulted in an approximate 80% decrease in cell viability after 18 h (Fig. 1B). In contrast, MDA-MB-231-wt and -mock cells displayed no significant differences in glucose deprivation-induced cell death (Fig. 1B). The increase in glucose deprivation-induced cell death by NDRG2 expression was also verified by FACS analysis of sub-G1 DNA content (Fig. 1C). An increase in cell death was further confirmed by western blot analysis, which showed cleavage of poly (ADP-ribose) polymerase (PARP) (Fig. 1D).

### NDRG2 overexpression attenuates glucose deprivation-induced AMPK activity in MDA-MB-231 cells

**Fig. 2:**
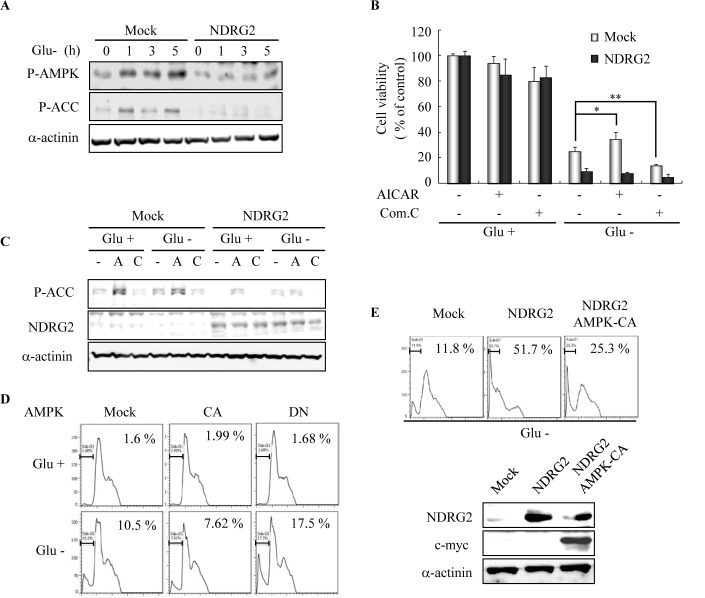
NDRG2 overexpression attenuates glucose deprivation-induced AMPK activity (A) MDA-MB-231-mock and -NDRG2 cells were exposed to glucose deprivation for indicated times. Total cell extracts were prepared and subjected to western blot analysis using specific antibodies. (B and C) The cells were pretreated with 1 mM AICAR (A) or 10 mM compound C (C) for 30 min and then exposed to glucose deprivation for 18 h. Cell viability was examined with the MTT assay. The results of three experiments are expressed as the mean ± SE (*p < 0.05 and **p < 0.01). After 3 h of glucose deprivation, total cell extracts were prepared and subjected to western blot analysis using specific antibodies. (D) MDA-MB-231 cells were transfected with plasmids encoding c-myc-tagged AMPKα -CA (constitutively active form) and AMPKα -DN (dominant negative form) and exposed to glucose deprivation for 24 h. The cells were analyzed for apoptosis by FACS analysis. The percentage of cells with sub-G1 DNA content is indicated. (E) MDA-MB-231 cells were manipulated to co-express both the CA form of AMPK and NDRG2 and then exposed to glucose deprivation for 36 h. Cell death in these cells was assessed by analyzing the percentage of cells with sub-G1 DNA content. To confirm the co-expression of NDRG2 and AMPK-CA, western blot analysis was performed.

Glucose deprivation in the solid tumor microenvironment results in an increase in the AMP:ATP ratio and the subsequent activation of AMPK [23]. We addressed whether AMPK was associated with an increase in glucose deprivation-induced cell death upon NDRG2 expression. Glucose deprivation markedly increased the phosphorylation of Thr^172^ in the catalytic subunit of AMPK and the phosphorylation of Ser^79^ in the AMPK substrate acetyl-CoA carboxylase (ACC) (Fig. 2A) [16]. In contrast, the AMPK activity that was induced by glucose deprivation was strongly inhibited in a time-dependent manner by NDRG2 overexpression. As a result, NDRG2 overexpression reduced glucose deprivation-induced AMPK activity in MDA-MB-231 cells. To evaluate the role of AMPK activity in glucose deprivation-induced cell death, we used pharmacological regulators of AMPK. The inhibition of AMPK activity by the AMPK inhibitor compound C resulted in a significant increase in glucose deprivation-induced cell death (Fig. 2B). In contrast, the effect of compound C was marginal in cells that were exposed to the normal medium and the activation of AMPK by the AMPK activator AICAR decreased glucose deprivation-induced cell death. However, glucose deprivation-induced cell death in NDRG2-overexpressing cells was not significantly affected by AMPK activator or inhibitor treatment because NDRG2 overexpression also attenuated AICAR-induced AMPK activity (Fig. 2C). To support these results, we used a molecular approach to regulate AMPK activity. As shown in Fig. 2D, the inhibition of AMPK activity by expression of a dominant-negative (DN) form of AMPK resulted in a marked increase in glucose deprivation-induced cell death. In contrast, the effect of the DN form of AMPK was marginal in cells that were exposed to normal medium and the activation of AMPK activity by expression of a constitutively active (CA) form of AMPK decreased glucose deprivation-induced cell death.

To examine the causal relationship between AMPK and NDRG2-induced apoptosis under conditions of glucose deprivation, we co-expressed NDRG2 and the CA form of AMPK in MDA-MB-231 cells and assessed the sub-G1 DNA content. NDRG2 expression induced glucose deprivation-induced apoptosis more effectively than the mock-control, and the expression of the CA form of AMPK significantly abrogated NDRG2-induced apoptosis under conditions of glucose deprivation (Fig. 2E). These data indicate that the inhibition of glucose deprivation-induced AMPK activity by NDRG2 overexpression renders tumor cells with an increased sensitivity to cell death after glucose deprivation.

### NDRG2 overexpression increases 2-DG-induced apoptosis

**Fig. 3:**
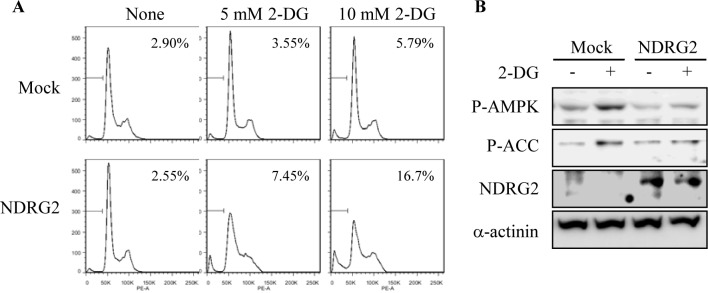
NDRG2 overexpression increases 2-DG-induced apoptosis (A) MDA-MB-231-mock and -NDRG2 cells were treated with the indicated concentrations of 2-DG for 48 h and then analyzed for apoptosis using FACS analysis. (B) Both cell lines were incubated with 2 mM 2-DG for 1 h. Total cell extracts were prepared and subjected to western blot analysis using specific antibodies.

The non-metabolizable glucose analog 2-DG is a specific blocker of glycolysis and mimics the effects of energy starvation [24]. The effects of 2-DG may be attributed to ATP depletion, which induces the activation of AMPK. To complement the results of experiments with glucose deprivation, we investigated the effects of NDRG2 overexpression in 2-DG-treated MDA-MB-231 cells. Whereas 2-DG increased apoptosis in both MDA-MB-231-mock and -NDRG2 cells in a dose-dependent manner, its efficacy as an inducer of apoptosis was higher in NDRG2-overexpressing cells than in the mock cells (Fig. 3A). Furthermore, although 2-DG treatment markedly enhanced AMPK activity, the activity was significantly inhibited by NDRG2 overexpression (Fig. 3B). These data indicate that the inhibition of AMPK activity by NDRG2 overexpression sensitizes MDA-MB-231 cells to 2-DG-induced apoptosis.

### NDRG2 overexpression increases hypoxia-induced apoptosis

**Fig. 4:**
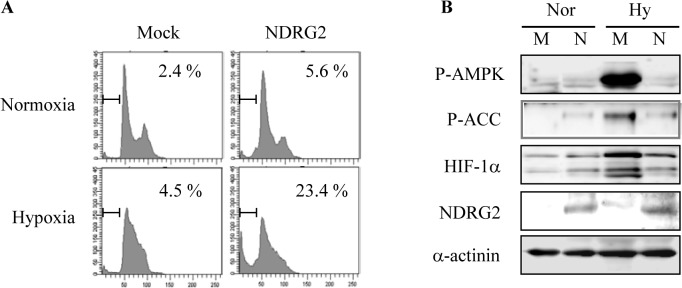
Effect of NDRG2 overexpression on hypoxia-induced apoptosis (A) MDA-MB-231-mock and -NDRG2 cells were incubated under normoxic or hypoxic conditions (1% O_2_, 72 h). Cells were collected, fixed in 70% ethanol, and stained with propidium iodide before FACS analysis. The percentage of cells with sub-G1 DNA content is indicated. The data from two experiments are expressed as the mean ± SE. (B) MDA-MB-231-mock (M) and -NDRG2 (N) cells were incubated under normoxic (Nor) or hypoxic (Hy) conditions for 2 h. Total cell extracts were prepared and subjected to western blot analysis using specific antibodies.

Hypoxia is another characteristic feature of the solid tumor microenvironment and represents one of the critical factors that are associated with drug resistance in most solid tumors. Hypoxia-induced AMPK activity protects cancer cells against hypoxic stress [25]. Therefore, we investigated whether NDRG2 overexpression increased hypoxia-induced apoptosis and prevented hypoxia-induced AMPK activity. Apoptosis using FACS analysis of sub-G1 DNA content was slightly induced when cells were exposed to hypoxia (1% O_2_) alone for 48 h. In contrast, NDRG2 overexpression strongly increased hypoxia-induced apoptosis in MDA-MB-231 cells (Fig. 4A). NDRG2 overexpression also markedly reduced hypoxia-induced AMPK activity and HIF-1 levels (Fig. 4B). These data support the notion that the inhibition of AMPK activity by NDRG2 overexpression sensitizes MDA-MB-231 cells to hypoxia-induced apoptosis.

### LKB1 is required for the inhibition of AMPK signaling by NDRG2

**Fig. 5:**
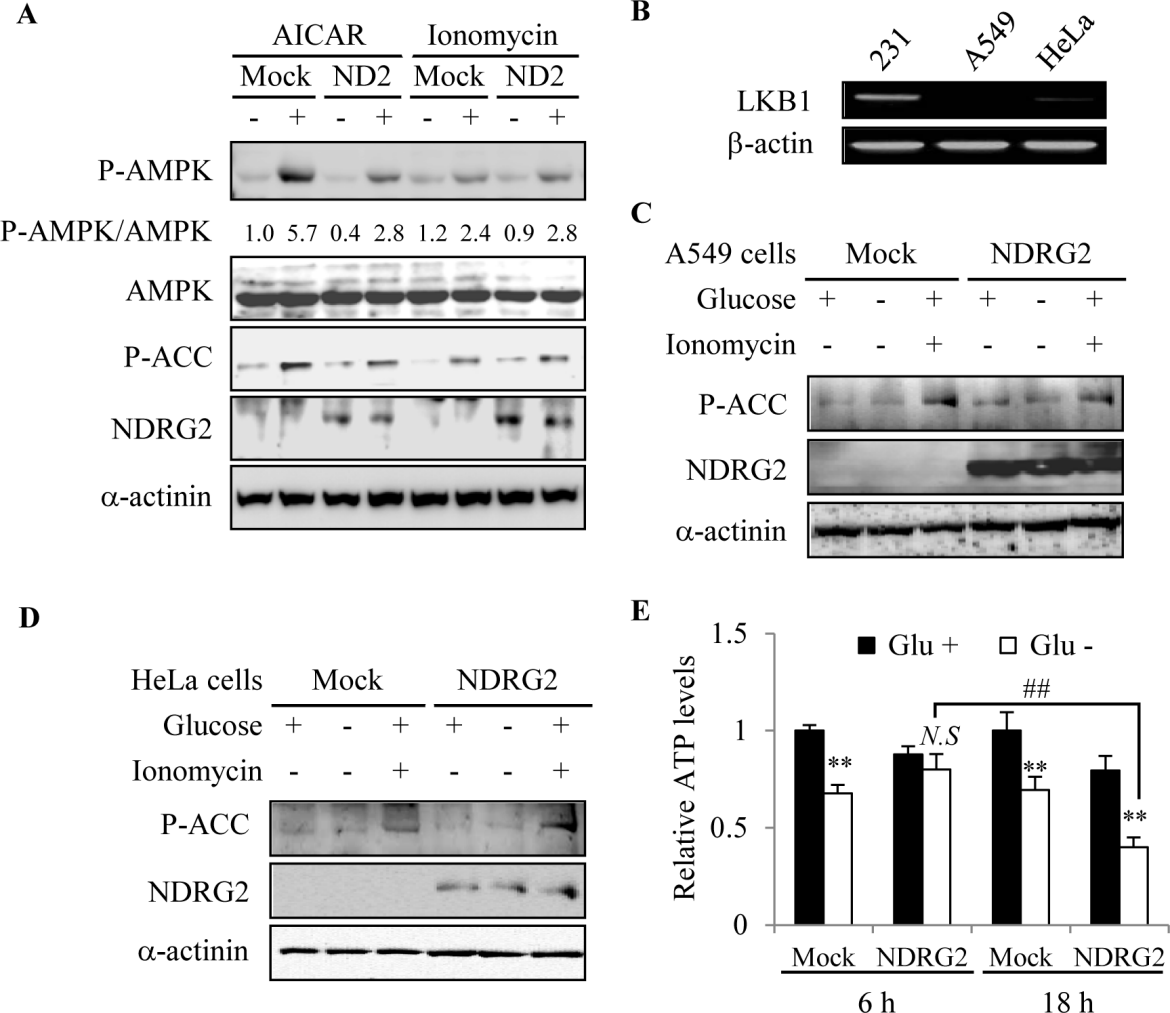
LKB1 is required for the inhibition of AMPK signaling by NDRG2 (A) MDA-MB-231-mock and -NDRG2 (ND2) cells were treated with 1 mM AICAR (1 h) or 1 μM ionomycin (15 min). Total cell extracts were prepared and subjected to western blot analysis using specific antibodies. Densitometry was used to determine the fold induction. (B) The mRNA levels of LKB1 and β -actin in MDA-MB-231, A549, and HeLa cells were compared by RT-PCR. (C and D) A549 and HeLa cells were transfected with the NDRG2 expression vector and then treated with 1 μM ionomycin (15 min) or incubated under conditions of glucose deprivation (1 h). Total cell extracts were prepared and subjected to western blot analysis using specific antibodies. (E) Both cell lines were exposed to glucose deprivation for the indicated times. Intracellular ATP levels were then assessed via the luciferin/luciferase method using an ATP determination kit. The data from two experiments are expressed as the mean ± SE (*N.S*, not significant, ^##^p < 0.01 and **p < 0.01).

The activation of AMPK, which is defined by the phosphorylation status of Thr^172^, involves the two major upstream kinases, LKB1 and CaMKK2. To understand the mechanisms of the NDRG2-induced inhibition of AMPK signaling, we attempted to examine AMPK activators that are known to activate AMPK via distinct upstream kinases. AICAR and ionomycin are known to induce AMPK activation via the upstream kinases, LKB1 and CaMKK2, respectively [26]. Interestingly, when MDA-MB-231-mock and -NDRG2 cells were exposed to 1 mM AICAR for 1 h or to 1 μM ionomycin for 15 min, we found that NDRG2 overexpression strongly inhibited AICAR-induced AMPK activity in MDA-MB-231 cells but did not affect ionomycin-induced AMPK activity (Fig. 5A). To further confirm whether the regulation of AMPK activity by NDRG2 overexpression is indeed associated with LKB1, we examined AMPK activation in LKB1-deficient HeLa cervical cancer cells and A549 lung adenocarcinoma cells. As expected, LKB1 was not detectable in the two cell lines (Fig. 5B). When HeLa and A549 cells were stimulated by glucose-deprivation or with ionomycin, ionomycin but not glucose deprivation markedly stimulated the Ser^79^ phosphorylation of ACC in A549 cells (Fig. 5C). Similar results were observed in HeLa cells, and the results were not affected by NDRG2 overexpression (Fig. 5D). We next determined the levels of intracellular ATP in both MDA-MB-231-mock and -NDRG2 cells that were exposed to glucose deprivation for 6 or 18 h. Glucose deprivation reduced the intracellular ATP level in MDA-MB-231-mock cells. However, whereas the intracellular ATP level was dramatically reduced after 18 h of glucose deprivation in MDA-MB-231-NDRG2 cells, a 6 h glucose deprivation did not affect the intracellular level of ATP (Fig. 5E). These data indicate that glucose deprivation induces AMPK activity through the LKB1 pathway and that NDRG2 overexpression reduces the activity of the LKB1-AMPK axis by blocking the reduction of intracellular ATP levels during the early phase of the response to glucose deprivation.

### NDRG2 overexpression enhances glucose deprivation- and hypoxia-induced apoptosis in HCT116 colon cancer cells

**Fig. 6:**
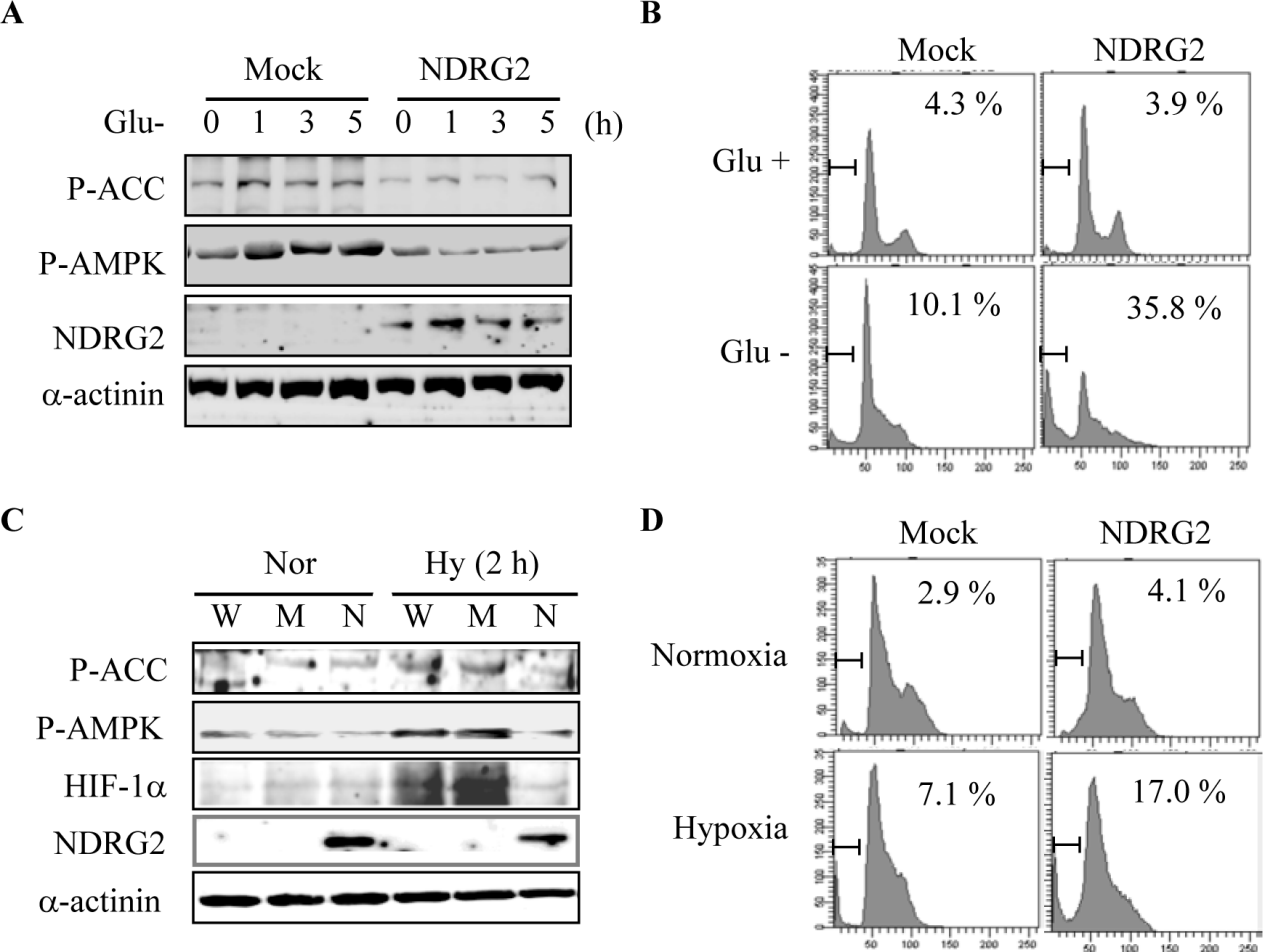
NDRG2 overexpression enhances glucose deprivation- and hypoxia-induced apoptosis in HCT116 colon cancer cells (A) HCT116 cells were transfected with an empty vector or a vector encoding NDRG2 gene. The cells were then exposed to glucose deprivation for the indicated times. Total cell extracts were prepared and subjected to western blot analysis using specific antibodies. (B) HCT116 cells that were transfected with an empty vector or a vector encoding NDRG2 were exposed to glucose deprivation for 24 h. The cells were collected, fixed in 70% ethanol, and stained with propidium iodide before FACS analysis. The percentage of cells with sub-G1 DNA content is indicated. (C) HCT116 cells that were transfected with an empty vector or with a vector encoding NDRG2 were incubated under normoxic or hypoxic conditions (1% O_2_) for 2 h. Total cell extracts were prepared and subjected to western blot analysis using specific antibodies. (D) After 48 h of hypoxic conditions, the cells were collected, fixed in 70% ethanol, and stained with propidium iodide before FACS analysis. The percentage of cells with sub-G1 DNA content is indicated.

To further confirm that NDRG2 overexpression regulates AMPK activity in other cancer cell types, we investigated the effects of NDRG2-induced inhibition of AMPK activity in HCT116 colorectal carcinoma cells, which were previously used to show the anticancer effect of NDRG2 expression [27]. Glucose deprivation markedly increased AMPK phosphorylation on Thr^172^ in the catalytic subunit and ACC phosphorylation on Ser^79^ in HCT116 cells. In contrast, NDRG2 overexpression strongly inhibited glucose deprivation-induced AMPK activation in a time-dependent manner (Fig. 6A). Glucose deprivation-induced apoptosis was also greatly enhanced by NDRG2 overexpression in HCT116 cells (Fig. 6B). We next investigated whether NDRG2 overexpression increases hypoxia-induced apoptosis and prevents hypoxia-induced AMPK activation in HCT116 cells. NDRG2 overexpression also suppressed hypoxia-induced AMPK activation and HIF-1 expression (Fig. 6C). Consistent with the AMPK inhibition model, NDRG2 overexpression significantly increased the level of hypoxia-induced apoptosis in HCT116 cells (Fig. 6D). Collectively, these data indicate that NDRG2 overexpression regulates AMPK activation and cell apoptosis in MDA-MB-231 and HCT116 cells in a similar manner.

## DISCUSSION

The present study investigated whether an association between NDRG2 expression and the intracellular energy regulator AMPK plays a role in tumor cell apoptosis. Our data clearly demonstrated that NDRG2 overexpression effectively reduced glucose deprivation-induced AMPK activity and increased glucose deprivation-induced apoptosis.

NDRG2 is a novel tumor suppressor gene and may play an important role in cancer. NDRG2 mRNA and protein levels have been shown to be down-regulated in a variety of human cancer cell lines and tumor tissues [2]. CpG island methylation, which changes NDRG2 gene expression patterns, has been observed in breast [28], colon [29], and lung cancer cells [28]. NDRG2 also functions as a prognostic marker in gastric cancer [30]. The anti-cancer effect of NDRG2 is associated with many important signaling pathways in a variety of human cancers. In our previous study using colon carcinoma cells, NDRG2 modulated the cell cycle via phosphorylation of c-Jun and the down-regulation of AP-1 and cyclin D1 [8]. NDRG2 also suppresses matrix metalloproteinase-9 (MMP-9) expression through the induction of BMP-4 secretion [31] and inhibits NF-kappaB activity and MMP-2 and -9 secretion [7], which abrogates the metastatic potential of breast cancer and fibrosarcoma cells. Moreover, NDRG2 expression negatively regulates JAK2/STAT3 through the regulation of the suppressor of cytokine signaling 1 gene in breast cancer cells [32]. Although evidence for the anti-cancer effects of NDRG2 is expanding, the functions of NDRG2 in metabolism field remain uncertain. In the present study, the involvement of AMPK signaling in the NDRG2-realated pathway which was a previously unknown signaling pathway was demonstrated. NDRG2 expression was shown to attenuate glucose deprivation-induced AMPK activity and to perform a critical function in glucose deprivation-induced cell death.

AMPK is a pivot point between cell survival and apoptosis, and it is a novel therapeutic target for metabolic diseases such as cancer [33-35]. A number of studies have reported that AMPK exerts pro-apoptotic influences on cancer. AMPK function is mediated in part by tumor suppressor proteins that are associated with the AMPK signaling pathway, including LKB1 [36], p53 [37], and tuberous sclerosis complex 2 [38]. However, AMPK also plays a central role in the cellular adaptation to ATP-depleting stress, such as glucose deprivation [12]. Once AMPK is activated, ATP consuming pathways are inhibited and ATP-generating pathways are stimulated. Therefore, AMPK optimizes total cellular ATP levels and protects cells from the stress of ATP depletion. Several studies have recently revealed that AMPK activation protects tumor cells from cell death that is triggered by chemotherapy [16] or glucose deprivation [21]. Within certain parts of the immune system, autophagy that is induced by the T cell immunoglobulin- and mucin domain-containing molecule-4-AMPK pathway in phagosomes also attenuates anti-tumor effects following chemotherapy [39]. AMPK also performs a key function in hypoxia-induced glycolysis and survival [25,40]. Thus, the extremely intricate regulatory mechanisms that involve AMPK exist for monitoring cellular energy levels under conditions of metabolic stress and for regulating cellular decisions either to undergo apoptosis or to survive. When conducting drug development studies that are focused on AMPK-related signaling, the multifaceted properties of AMPK demand a deeper understanding of how AMPK regulates the microenvironment of solid tumors. In our study, glucose deprivation and hypoxia, which represent the microenvironment of solid tumors, induced AMPK activation through the LKB1 pathway. This activity also plays a critical role in tumor survival. Moreover, NDRG2 overexpression attenuates glucose deprivation- and hypoxia-induced AMPK activity and increases glucose deprivation- and hypoxia-induced cell death in the LKB1 positive cell lines MDA-MB-231 and HCT116. We speculate a role for AMPK that is tightly associated with AMPK's central functions that regulate energy homeostasis.

Anoikis is particularly crucial for the metastasis of cancers [41]. If cancer cells do not die after detachment from the primary site, these cells are displaced from the matrix component and then move through the bloodstream to a secondary site. Anoikis is therefore an important mechanism by which metastasis occurs with dispatch. Numerous studies support the anti-metastatic potential of NDRG2 [2,7,31,42,43]. Cancer cells also exhibit altered energy metabolism and resistance to anoikis via the regulation of AMPK activity [44,45]. Although the results of our work (Fig. 1B and C) reveal similar patterns in terms of an enhancement of the glucose deprivation-induced cell death by NDRG2 expression, different results were obtained with the MTT and FACS assays (sub-G1 DNA content). NDRG2 overexpression along with glucose deprivation resulted in an approximate 80% reduction in cell viability in the MTT assay. However, the percentage of cells with sub-G1 DNA content (15.6%) indicated a lower reduction in cell death than the MTT assay. Because the MTT assay is based on the use of adherent cells, this assay does not distinguish the live cells that are resistant to anoikis from the cells that remain in suspension. Therefore, we speculate that NDRG2 overexpression may reduce the resistance to anoikis via the inhibition of glucose deprivation-induced AMPK activity.

In conclusion, the results of this study identified a major role for NDRG2 in increasing glucose deprivation-induced cell death through inhibitory effects on AMPK activity. Our work also identified that NDRG2 exerts anti-cancer effects under conditions that mimic the microenvironment of solid tumors, which include glucose deprivation and hypoxia. Furthermore, the function of LKB1 as an upstream kinase of AMPK was corroborated by the observation that NDRG2 overexpression did not significantly affect inhibition of the glucose deprivation-induced AMPK activity in HeLa and A549 cells that lack LKB1. Our study also suggests that the difference in NDRG2 expression in tumor cells may result in differential responses to micro-environmental stimuli and may influence tumor progression and patient prognosis, which suggests that NDRG2 may be developed as a clinically relevant molecular biomarker that may potentially be used in cancer diagnosis and therapy.

## MATERIAL AND METHOD

### Materials

Dulbecco's modified Eagle's medium (DMEM), glucose-free DMEM, and fetal bovine serum (FBS) were obtained from Gibco/Invitrogen (Carlsbad, CA). Propidium iodide and 3-[4,5-dimethylthiazol-2-thiazolyl]-2,5-diphenyltetrazolium bromide (MTT) were obtained from Sigma-Aldrich (St. Louis, MO). 5-Aminoimidazole-4 carboxaminde-1-beta-D-ribofuranoside (AICAR) and antibodies recognizing the phospho-specific forms of AMPKα -Thr^172^ and acetyl-CoA carboxylase (ACC)-Ser^79^ were purchased from Cell Signaling Technology (Boston, MA). Antibodies against α -actinin, NDRG2, myc, poly (ADP-ribose) polymerase (PARP), and AMPKα were purchased from Santa Cruz Biotechnology (Santa Cruz, CA). An antibody against HIF-1α was purchased from Bethyl Laboratories, Inc. (Montgomery, TX). Compound C was purchased from Calbiochem (San Diego, CA).

### Cell culture and hypoxia

MDA-MB-231 (breast adenocarcinoma), HCT116 (human colon carcinoma), HeLa (human cervix adenocarcinoma), and A549 (human lung carcinoma) cells (ATCC, Manassas, VA) were maintained in DMEM supplemented with 10% heat-inactivated FBS and antibiotics at 37 °C with 95% air and 5% CO_2_. The culture medium was removed and replaced before cells were exposed to hypoxic conditions. The dishes were transferred to an anaerobic chamber that was flushed with 1% O_2_, 5% CO_2_, and 94% N_2_ at 37 °C and were then incubated for the indicated periods of time.

### Overexpression of the NDRG2 gene in MDA-MB-231 cells and plasmid transfections

MDA-MB-231 cells were transfected with pCMV-Taq-2B-NDRG2 using WelFect-EX™ PLUS Transfection Reagent (WelGENE, Daegu, Republic of Korea). Stable cells were selected using complete medium containing 1 mg/ml neomycin (G418, Gibco/Invitrogen, Carlsbad, CA), and NDRG2 expression was confirmed by reverse transcription-PCR (RT-PCR) and western blotting. The human NDRG2 DNA was cloned into the pCMV-Taq-2B vector as described previously [7]. The c-myc-tagged dominant-negative (DN) and constitutively active (CA) forms of AMPK were prepared as described previously [46]. Plasmids were transfected into cells using PolyFect transfection reagent (Qiagen, Valencia, CA) according to the manufacturer's instructions.

### Cell viability assays and flow cytometric analysis of apoptosis

Cell apoptosis was assessed using a fluorescence-activated cell sorter (FACS). NDRG2-modified cells were exposed to glucose-free medium for 18 or 24 h. Cells were harvested by trypsinization and washed with PBS. After fixation in 70% ethanol, the cells were resuspended in PBS that contained 10 μg/ml propidium iodide. The fluorescence intensity was determined using a FACSCanto™II flow cytometer (BD Biosciences, Carlsbad, CA). Cell viability was assessed using the MTT assay. The cells were treated with 5 μ g/ml MTT solution and then incubated for 2 h. They were dissolved in DMSO, and the absorbance was measured at 570 nm.

### Western blotting

The treated cells were lysed on ice in PRO-PREP™ Protein Extraction Solution (iNtRON Biotechnology, Seoul, Korea) for 30 min at 4 °C to prepare the whole-cell lysates. The supernatant fractions were recovered by centrifugation (14,000 × g, 20 min, 4 °C), and the protein concentrations of the lysates were determined using a Bradford protein assay. Samples were prepared with 2-mercaptoethanol and denatured by heating at 95 °C for 3 min. The proteins were separated on 8-12% polyacrylamide gels and transferred to nitrocellulose membranes. The membranes were blocked with 1% bovine serum albumin or 5% skim milk and hybridized with the primary antibody. The protein bands were visualized using a chemiluminescence detection kit (Amersham Pharmacia Biotech, Piscataway, NJ) and a LAS-3000 or LAS-4000 imaging system (FUJIFILM Corporation, Tokyo, Japan) after hybridization with the HRP-conjugated secondary antibody. The band intensities of the western blot data were analyzed using Quantity One software (Bio-Rad Laboratories, Hercules, CA).

### RNA extraction and reverse transcription PCR (RT–PCR)

Total RNA was extracted from the cells using TRIzol reagent (Invitrogen, Carlsbad, CA) based on the manufacturer's instructions and was reverse transcribed to complementary DNA using M-MLV reverse transcriptase (Promega, Madison, WI) and oligo (dT) primers. c DNA aliquots of 5 μg RNA were analyzed using semi-quantitative PCR. The PCR products were electrophoresed on 1% agarose gels containing ethidium bromide.

### ATP analysis

Intracellular ATP was extracted from cells and measured by the luciferin/luciferase method with the ATP Determination Kit (Molecular Probes). The assay buffer (100 μ l), which contained 0.5 mM luciferin, 1.25 μ g/ml luciferase, 25 mM Tris pH 7.8, 5 mM MgSO_4_, 100 μM EDTA, and 1 mM DTT, was mixed with 20 μ l of the cell lysate. Luminescence was analyzed and normalized using the cellular protein level.

### Statistical analysis

The results are presented as the means ± SD. All experiments were repeated at least three times, and the data were analyzed for statistical significance using GraphPad Prism 5 software (GraphPad Software, La Jolla, CA). Significant differences were analyzed using one-way ANOVA tests that were followed by either a Newman-Keuls multiple comparison test if there were more than three groups or an unpaired *t*-test if there were only two groups. *P* values less than 0.05 were considered significant.
